# A novel ^11^C-labeled thymidine analog, [^11^C]AZT, for tumor imaging by positron emission tomography

**DOI:** 10.1186/s13550-015-0124-0

**Published:** 2015-09-04

**Authors:** Tsuyoshi Tahara, Zhouen Zhang, Masahiro Ohno, Yukako Hirao, Nami Hosaka, Hisashi Doi, Masaaki Suzuki, Hirotaka Onoe

**Affiliations:** Division of Bio-Function Dynamics Imaging, RIKEN Center for Life Science Technologies (CLST), 6-7-3 Minatojima, Minamimachi, Chuo-ku, Kobe, Hyogo 650-0047 Japan; RIKEN Center for Molecular Imaging Science, Kobe, Japan

**Keywords:** AZT, d4T, PET, Thymidine analog, Tumor imaging

## Abstract

**Background:**

Nucleoside analogs labeled with positrons, such as ^11^C and ^18^F, are considered valuable in visualizing the proliferative activity of tumor cells in vivo using positron emission tomography (PET). We recently developed the ^11^C-labeled thymidine analogs [^11^C]zidovudine ([^11^C]AZT) and [^11^C]stavudine ([^11^C]d4T) via the Pd(0)-Cu(I) co-mediated rapid C–C coupling reaction. In this study, to examine whether [^11^C]AZT and [^11^C]d4T might be useful for visualization of tumors in vivo, we performed PET imaging, tissue distribution studies, and metabolite analysis in tumor-bearing mice.

**Methods:**

Mice bearing tumors (rat glioma C6 and human cervical adenocarcinoma HeLa cells) were injected with 50 MBq of [^11^C]AZT or [^11^C]d4T, and PET was performed immediately thereafter. After PET imaging, the radioactivity in several tissues, including tumor tissues, was measured using a γ-counter. In addition, radioactive metabolites in plasma, bile, intestinal contents, and tumor were analyzed using thin layer chromatography (TLC). Cellular uptake of [^11^C]AZT in C6 was measured in the presence or absence of non-labeled thymidine (0.1 mM).

**Results:**

In PET studies, C6 and HeLa tumors in mice were clearly visualized using [^11^C]AZT. Time-activity curves using [^11^C]AZT showed that the accumulation of radioactivity in tumors plateaued at 10 min after injection and persisted for 60 min, while most of the radioactivity in other tissues was rapidly excreted into the urine. In various tissues of the body, tumor tissue showed the highest radioactivity at 80 min after injection (five to six times higher uptake than that of blood). Compared with tumor tissue, uptake was lower in other proliferative tissues such as the spleen, intestine, and bone marrow, resulting in a high tumor-to-bone marrow ratio. Cellular uptake of [^11^C]AZT in C6 cells was completely blocked by the application of thymidine, strongly indicating the specific involvement of nucleoside transporters. In contrast, the time-activity curve of [^11^C]d4T in the tumor showed transient and rapid excretion with almost no obvious tumor tissue accumulation.

**Conclusions:**

Tumors can be detected by PET using [^11^C]AZT; therefore, [^11^C]AZT could be useful as a novel PET tracer for tumor imaging in vivo.

**Electronic supplementary material:**

The online version of this article (doi:10.1186/s13550-015-0124-0) contains supplementary material, which is available to authorized users.

## Background

Thymidine analog nucleoside reverse transcriptase inhibitors (NRTIs) such as zidovudine (AZT) and stavudine (d4T) suppress the replication of human immunodeficiency virus (HIV) and are now used in the treatment of acquired immunodeficiency syndrome (AIDS) [1, 2]. Although AZT was the first anti-HIV drug to be approved worldwide, it was originally designed as an antitumor agent due to its prevention of DNA elongation by inhibiting the incorporation of thymidine into the DNA of cancer cells [3, 4].

Increased cell proliferative activity is a prominent feature of tumor cells. The capacity to non-invasively measure this proliferative activity is a potent tool in the clinical detection and diagnosis of tumors. In order to measure tumor proliferative activity using positron emission tomography (PET), several thymidine analogs have been labeled with positron-emitting nuclei, such as ^11^C and ^18^F [5–11]. One of the most promising PET tracers for proliferation imaging is 4′-[methyl-^11^C]thiothymidine ([^11^C]4DST), which is resistant to degradation by thymidine phosphorylase, and can be incorporated into DNA [5]. Although [^11^C]4DST has shown high accumulation in tumors in mouse models and in patients [5, 12, 13], concomitant accumulation occurred in normal tissues, such as the bone marrow, spleen, thymus, and intestine, because of their relatively high proliferative activity.

**Fig. 1:**
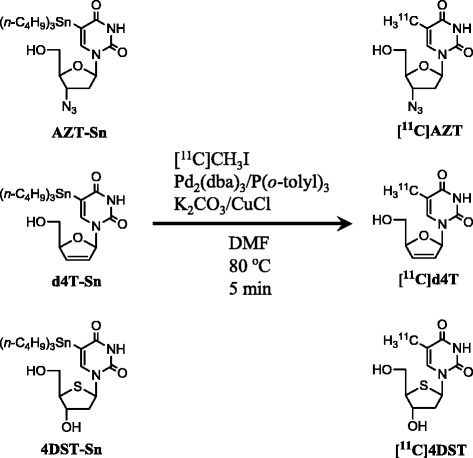
Scheme showing the synthesis of [^11^C]zidovudine (AZT), [^11^C]stavudine (d4T), and 4′-[methyl-^11^C]thiothymidine ([^11^C]4DST) [14]

We recently succeeded in the ^11^C-labeling of two anti-HIV drugs, to produce [^11^C]AZT and [^11^C]d4T, via the Pd(0)-Cu(I) co-mediated rapid C-C coupling reaction (Fig. 1) [14]. These labeled drugs may have potential roles as PET probes, not only in tumor diagnosis, but also in the evaluation of AIDS/HIV infection. In the present study, to evaluate whether these novel ^11^C-labeled probes might be useful in PET tumor imaging, we performed the following studies: (1) tissue distribution of the probes on PET imaging of C6 and HeLa tumor-bearing mice; (2) metabolite analysis by thin-layer chromatography (TLC) of plasma, bile, and tumor samples; and (3) a [^11^C]AZT cellular uptake study with non-labeled thymidine.

## Methods

### Reagents

Silica-gel RP-18 F254s TLC plates were purchased from Merck (Darmstadt, Germany) and 1.0-ml syringes were purchased from Terumo (Tokyo, Japan). Two AZT metabolites, 3′-Azido-3′-deoxythymidine 5′-monophosphate sodium salt and 3′-Azido-3′-deoxythymidine β-d-glucuronide sodium salt were purchased from Carbosynth Limited (Berkshire, UK) and Toronto Research Chemicals (Toronto, Canada), respectively. All other chemicals were purchased from Wako (Tokyo, Japan) or Nacalai Tesque (Kyoto, Japan) and were of analytical grade.

### Synthesis of [^11^C]AZT, [^11^C]d4T and [^11^C]4DST

[^11^C]Carbon dioxide was produced by a ^14^N(p,α)^11^C reaction using a CYPRIS HM-12S cyclotron (Sumitomo Heavy Industries, Tokyo, Japan), and was then converted into [^11^C]CH_3_I by LiAlH_4_ reduction followed by HI treatment, using an original automated synthesis system for ^11^C-labeling in RIKEN CLST. The obtained [^11^C]CH_3_I was used for the Pd(0)-Cu(I) co-mediated rapid [^11^C]methylation with precursors 3′-azido-2′,3′-dideoxy-5-(tributylstannyl)-uridine, 2′,3′-didehydro-2′,3′-dideoxy-5-(tributylstannyl)-uridine and 5-tributylstannyl-4′-thio-2′-deoxyuridine to generate the PET tracers [^11^C]AZT, [^11^C]d4T and [^11^C]4DST, respectively (Fig. 1) [14].

### Animal models

All experimental protocols were approved by the Animal Care and Use Committee of RIKEN Kobe Institute (MAH24-03) and were performed according to the guidelines for animal experiments published by the National Institutes of Health [15]. Rat glioma C6 and human cervix adenocarcinoma HeLa cells were grown in Dulbecco’s modified Eagle’s medium supplemented with 10 % fetal bovine serum and 100 μg/ml of penicillin-streptomycin. Five-week-old female BALB/c-nu/nu mice were obtained from CLEA Japan (Tokyo, Japan) and were held for 1 week prior to the experiment. Tumor-bearing mice were established by subcutaneous injection of tumor cells into the shoulder of mice (1 × 10^6^ cells for C6 and 5 × 10^6^ cells for HeLa), as described previously [16]. The mice were used for imaging experiments when the tumors had grown to approximately 5 to 10 mm in diameter.

For examination of tracer accumulation in the inflammatory area, mice were injected 0.03 ml of turpentine oil into the right thigh and were conducted to the PET imaging 3 days after the administration [17].

### MicroPET imaging

A small animal PET scanner (microPET Focus220, Siemens Medical Systems, Erlangen, Germany) was used for the imaging studies of the mice. A venous indwelling catheter was inserted into the tail vein for probe injection before PET scanning. Animals were kept on a heating pad to maintain a body temperature of 37 °C. The probes ([^11^C]AZT, [^11^C]d4T and [^11^C]4DST at a concentration of 50 MBq/0.1 ml) were injected under 1.5 % isoflurane anesthesia, and dynamic PET scans (6 × 10 s, 6 × 30 s, 11 × 60 s, and 15 × 180 s) were performed for 60 min, immediately after probe injection. For inflammation imaging with [^18^F]FDG (5 MBq/0.1 ml), mice fasted for 4 h before injection were kept for 50 min in awake state and then were anesthetized with 1.5 % isoflurane to image during 60–80 min after the injection. Emission data were acquired using a 3D list-mode method, and PET images were reconstructed using the filtered back projection algorithm. The maximum-intensity-projection images were displayed using ASIPro software (Concorde Microsystems, Knoxville, USA). The uptake of the probes in tissues is shown as standardized uptake value (SUV).

### Tissue distribution

A concentration of 50 MBq of probes in 100 μl of saline was injected into tumor-bearing mice via the tail vein. The mice were sacrificed under deep anesthesia with 3.0 % isoflurane at 80 min after the injection, and the blood, urine, brain, heart, lungs, liver, kidneys, spleen, pancreas, quadriceps muscles, ovaries, duodenum, intestinal contents, bone, and tumors were then collected, and their volumes and weights were promptly measured. The radioactivity in the various tissues, whole blood (WB), plasma, and urine samples was measured using a γ-well counter (Wizard 1480; PerkinElmer, Waltham, USA). The results are expressed as %ID/g [18].

### Thin layer chromatography

Metabolite analysis using thin layer chromatography (TLC) was performed for plasma, bile, intestinal contents, and tumor samples that were obtained at 30 or 60 min after probe injection. The blood sample was collected by cardiac puncture using a heparinized syringe under deep anesthesia with 3.0 % isoflurane. Subsequently, the blood was centrifuged at 16,000×*g* at 4 °C for 2 min, and the plasma (approximately 100 μl) was vortexed with an equal volume of acetonitrile. After centrifugation at 16,000×*g* at 4 °C for 2 min, the supernatant was collected as the TLC sample. To prepare TLC samples from the liver and tumor, weighed tissues were added to an equal volume (*w*/*v*) of distilled water, and were homogenized and centrifuged. The supernatants were vortexed with three volumes (*v*/*v*) of acetonitrile and were then centrifuged. The supernatants were collected and used as TLC samples. Aliquots of the acetonitrile-treated supernatants (2.0 μl) were then spotted on a TLC plate Silica-gel 60 RP-18 and were developed with the solvent (CH_3_CN/phosphate buffer (pH 6.8) = 15:85) until the front line reached 7 cm from the origin. The TLC plate was then placed on an imaging plate (BAS-SR2040; Fuji Photo Film Co., Tokyo, Japan) for 1 h, and the exposed imaging plate was scanned with a fluoro-image analyzer (FLA-7000IR; Fuji Photo Film). The proportion of each [^11^C]AZT metabolite was calculated based on the radioactivity in each metabolite (photostimulable luminescence/mm^2^).

### [^11^C]AZT cell uptake study with thymidine

For the uptake study, C6 cells were seeded on 6-well plates at a density of 2 × 10^5^ cells/well for 24 h. Cellular uptake was initiated by adding [^11^C]AZT (27 nM = 1.4 MBq/well) in the presence or absence of non-labeled thymidine (0.1 mM), and the cells were incubated at 37 °C for 30 min. Uptake was terminated by adding ice-cold phosphate-buffered saline, and the cells were washed three times with this buffer. The cells were lysed with buffer (25 mM Tris-HCl, pH 7.4, 2.5 mM EDTA, 1 % Triton X-100), and the radioactivity of the lysate was measured using a γ-well counter. Cellular protein concentrations were determined by the Coomassie blue method [19] using bovine serum albumin as the standard. All experiments were performed in triplicate.

### Statistical analysis

Data are expressed as means ± SD groups were compared using one-way or repeated measure (RM) two-way analysis of variance (ANOVA), and the Bonferroni method was used for the post hoc multiple comparison procedure with a significant level of *p* < 0.05.

## Results

### PET imaging with [^11^C]AZT

**Fig. 2:**
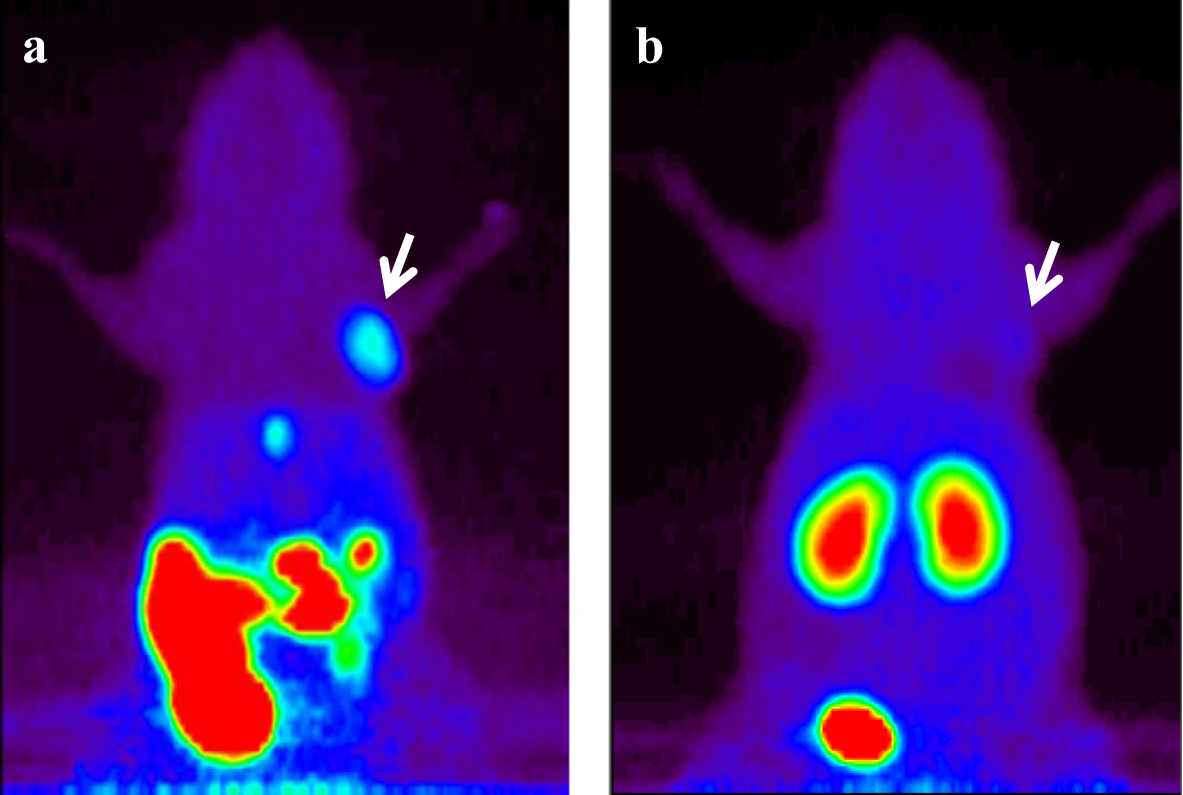
PET imaging of C6 tumor-bearing mice after injection of [^11^C]AZT or [^11^C]d4T. Projection PET images (60 to 80 min) of representative C6 tumor-bearing mice (n = 4) after injection of 50 MBq / 0.1 ml of [^11^C]AZT (**a**) or [^11^C]d4T (**b**) are shown. Arrows indicate the C6 tumors

**Fig. 3:**
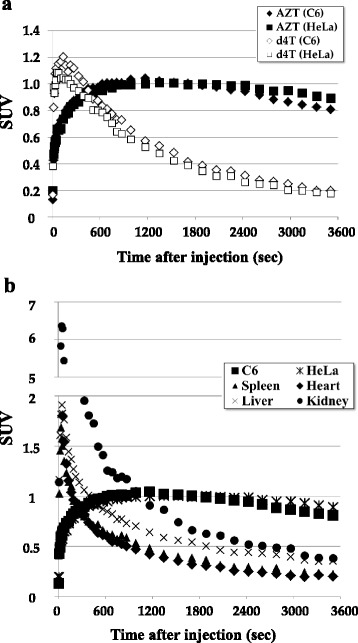
Time activity curves of injected [^11^C]AZT or [^11^C]d4T in the tissues of tumor-bearing mice. (**a**) Time changes of activity of [^11^C]AZT (expressed as Closed) or [^11^C]d4T (expressed as Opened) in C6 (expressed as Diamond) and HeLa (expressed as Square) tumor tissues over 3600 s. Statistical analysis of [^11^C]AZT injected mice vs [^11^C]d4T injected mice by RM two-way ANOVA indicates a significant difference, ***P < 0.001. (**b**) The uptake of [^11^C]AZT by tumor and several normal tissues over 3600 s after injection into C6 or HeLa tumor-bearing mice (n = 3 per group). The data were averaged from 3 mice and activity is indicated as the SUV

The whole-body summed PET images obtained between 60 and 80 min after injection of [^11^C]AZT clearly demonstrated high uptake in C6 tumors (Fig. 2a). This probe was detected not only in the mice bearing C6 tumors, but also in those bearing HeLa tumors (Additional file 1: Figure S1). In contrast, there was lower uptake of [^11^C]d4T in both C6 and HeLa tumors (Fig. 2b and Additional file 1: Figure S1, respectively). Time-activity curves (TACs) demonstrated that the radioactivity of [^11^C]AZT in both tumors plateaued at 10 min and was maintained at this level until 60 min after injection (Fig. 3a). There was no remarkable accumulation of [^11^C]AZT in normal tissues (e.g., heart, liver, spleen, and kidney) in both C6- and HeLa-bearing mice (Fig. 3b), but it did gradually accumulate in the urine over time on PET (data not shown). Additionally, we performed the PET studies with turpentine oil-induced inflammation model mice. [^11^C]AZT did not accumulate in inflammatory area (Additional file 1: Figure S2A-F).

### Tissue distribution of [^11^C]AZT in tumor-bearing mice

**Fig. 4:**
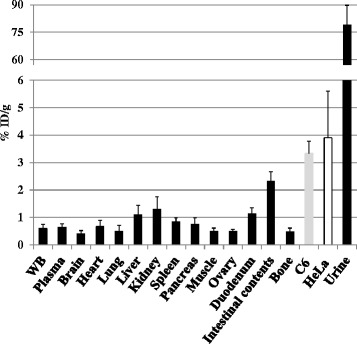
Tissue distribution of [^11^C]AZT in the tumor-bearing mice at 80 min after injection. The radioactivity in the indicated tissues, whole blood (WB), plasma, and urine samples of C6 (n = 4) and HeLa (n = 5) bearing mice was measured using a g-well counter at 80 min after injection of [^11^C]AZT. The results are presented as means ± S.D. of %ID / g. One way ANOVA; No significant

To investigate the tissue distribution of [^11^C]AZT in detail, we sacrificed the tumor-bearing mice at 80 min after injection and measured the radioactivity in the blood, urine, and several tissues including tumor tissues. Of the tissues tested, radioactivity levels were highest in the tumors (five to six times higher uptake than in blood), and radioactivity levels in the tumors were also higher than in other proliferative tissues such as the spleen, intestine, and bone marrow (Fig. 4). Since the TAC of [^11^C]d4T in the tumors showed transient and rapid excretion, with no observed accumulation as shown in Fig. 3a, the tissue distribution of [^11^C]d4T was not analyzed in further detail.

### Comparison of the accumulation of thymidine analogs in normal proliferative tissues of a C6 glioma-bearing mouse by PET analysis

**Fig. 5:**
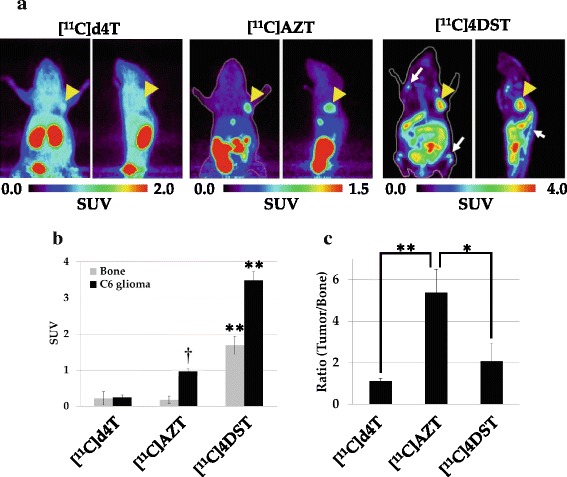
Comparisons of the *in vivo* accumulation level of thymidine analogs in C6 tumor-bearing mice. Summed PET images (60 to 80 min) of C6 tumor-bearing mice after injection of 50 MBq of [^11^C]AZT, [^11^C]d4T or [^11^C]4DST (**a**). The color code for the standardized uptake value (SUV) is shown at the bottom. Arrowheads indicate the C6 tumors. Arrows indicate bone or bone marrow regions. The graph in (**b**) shows the SUVs from PET images for tumor tissue and bone. Data are presented as means ± S.D. (n = 4 to 6). One way ANOVA;**P < 0.01 vs. [^11^C]AZT and [^11^C]d4T injected mouse. †*P* < 0.01 vs. [^11^C]d4T injected mouse. The graph in (c) shows the ratio of tumor-to-bone uptake of the labeled probes. One way ANOVA;*P < 0.05 vs. [11C]4DST injected mouse. **P < 0.01 vs. [^11^C]d4T injected mouse

To compare the differences in the pattern of accumulation of three ^11^C-labeled thymidine analogs in normal proliferative tissues in vivo, we performed PET analysis of injected C6 tumor-bearing mice under the same conditions and analyzed the ratio of tumor accumulation to bone accumulation for each analog in PET images. As shown in Fig. 5a, b and Additional file 1: Figure S3A, although [^11^C]4DST showed the highest tumor accumulation (3.5 times higher than the tumor accumulation of [^11^C]AZT), it was also highly accumulated in the bone. Thus, of the two thymidine analogs examined, [^11^C]4DST and [^11^C]AZT showed the lowest and highest tumor-to-bone ratios, respectively (Fig. 5c and Additional file 1: Figure S3B, *p* < 0.05).

### Metabolite analysis of [^11^C]AZT

**Fig. 6:**
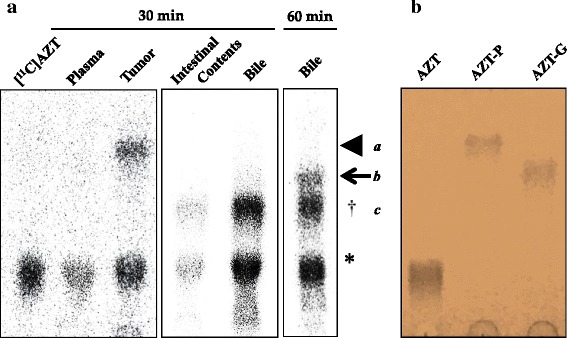
Metabolite analysis of injected [^11^C]AZT. **a** Representative TLC-radiochromatogram of plasma and tumor tissues at 30 min after *i.v.* injection and of the bile at 60 min after *i.v.* injection of [^11^C]AZT. * indicates unmetabolized [^11^C]AZT. *Arrowhead*, *arrow*, and † indicate the metabolites *a*, *b* and *c*, respectively. **b** The picture of TLC-chromatogram with unlabeled AZT metabolites visualized by ultraviolet (254 nm) irradiation

Metabolite analysis of plasma, bile, intestinal contents, and tumor at 30 or 60 min after the injection of [^11^C]AZT was then performed using TLC (Fig. 6a). Three major hydrophilic metabolites were detected: metabolites *a*, *b*, and *c.* To identify these metabolites, we carried out TLC with major AZT metabolites, AZT-5′-monophosphate (AZT-P) and AZT-β-d-glucuronide (AZT-G) (Fig. 6b). In plasma, the unmetabolized form of [^11^C]AZT constituted 93.3 ± 2.4 % of the total radioactivity; in the tumors, it accounted for approximately 50 % of the total radioactivity, with large amounts of metabolite *a* (45.6 ± 3.1 %) being present. The other metabolites, metabolite *b* and *c* were observed in the bile. The percentages of metabolites *b* and *c* in the bile were 9.2 ± 2.3 % and 39.6 ± 5.5 %, respectively. The R_*f*_ values of metabolite *a* and *b* were consistent with that of AZT-P and AZT-G, respectively (Fig. 6a, b).

### In vitro [^11^C]AZT uptake study using tumor cells

**Fig. 7:**
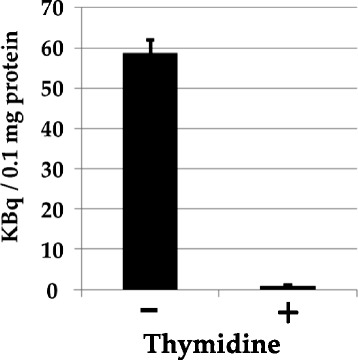
Effect of thymidine on [^11^C]AZT uptake by C6 cells. [^11^C]AZT (27 nM) uptake was measured after incubation of the cells at 37 °C for 30 min in the presence or absence of thymidine (0.1 mM). Data are averages of three independent experiments. Data are presented as means ± SD

To investigate whether nucleotide transporters are involved in [^11^C]AZT uptake in C6 tumors, we performed an uptake study using thymidine as a competitor. As shown in Fig. 7, the uptake of [^11^C]AZT into C6 cells was completely inhibited by the addition of non-labeled thymidine, suggesting that nucleotide transporters are involved in [^11^C]AZT uptake.

## Discussion

In this study, we successfully determined whether it was possible to measure the proliferative activity of tumors in a tumor-bearing mouse model by PET scanning using either of two novel PET ligands, [^11^C]AZT and [^11^C]d4T. The results revealed the usefulness of [^11^C]AZT for in vivo tumor imaging.

In previous studies, tumor imaging of thymidine analogs was used in order to assess their impact on cell proliferation [5–11]. Recently, Toyohara et al. reported that [^11^C]4DST was useful in imaging of tumor cell proliferation in vivo in mice and humans, because it was a substrate of the enzyme involved in the phosphorylation of thymidine but was resistant to degradation [5, 12, 13]. However, in those PET studies, [^11^C]4DST was also highly accumulated in normal proliferative tissues such as the bone marrow, spleen, and duodenum, which made it difficult to distinguish tumors from normal proliferative cells. In the present PET study of a mouse tumor model, as shown in Fig. 5, although the maximal SUV of [^11^C]AZT in tumor tissue was less than that of [^11^C]4DST, the accumulation of [^11^C]AZT in tumor tissue was much higher than that in the abovementioned normal proliferative tissues. In addition, [^11^C]AZT showed a higher tumor-to-bone marrow ratio than [^11^C]4DST. The mechanism that underlies this difference is unknown, but it may be related to the AZT substrate specificity of enzymes in the thymidine-phosphorylation pathway (thymidine kinase and thymidylate kinase), which are described in a later paragraph. Based on these data, [^11^C]AZT is expected to be useful for detecting tumors in bone or bone metastasis. Further studies are needed to validate this possibility using bone metastasis model animals.

Previous in vitro and in vivo studies have demonstrated that AZT is metabolized to AZT-*β*-d-glucuronide (AZT-G) by UDP-glucuronosyltransferase in hepatocytes and that it is excreted mainly into the urine in rats [20, 21], dogs [20], monkeys [20, 22], and humans [20–22]. In the present TLC analysis, although very little metabolism and degradation of [^11^C]AZT occurred in vivo in mice, we did observe three hydrophilic metabolites of [^11^C]AZT, two of which were observed in the bile (metabolite *b* and *c*, Fig. 6). Combined with the previous results [19–21], this finding suggests that the metabolite *b* found in the bile may be AZT-G because its R_*f*_ value showed the same position that of AZT-G (Fig. 6a, b). AZT-G in bile was observed at 60 min after the injection but not at 30 min while much amount of metabolite *c* was observed in bile and intestinal contents after 30 min at the lower position of AZT-G in TLC. Therefore, it is possible to consider that low liver background uptake may be derived by faster production of metabolite *c*, which excreted into urine or intestinal contents via the bile bladder and a little and delayed production of AZT-G in liver. Since the metabolite *c* could not identify in the present study, so it would need further study to clarify the form.

Although AZT is known to be phosphorylated to 5′-monophosphate by enzymes in the thymidine-phosphorylation pathway (thymidine kinase and thymidylate kinase), little AZT is converted to 5′-di- and 5′-tri-phosphate forms in humans [23], rats [24], and mice [25]. Therefore, the one major metabolite (metabolite *a*) that was observed in tumor tissues (Fig. 6) may be the 5′-monophosphate form of [^11^C]AZT (AZT-P). In Fig. 6a and b, TLC analysis with unlabeled compounds indicated that the R_*f*_ value of AZT-P closely resemble that of metabolite *a*. Furthermore, these data suggest that [^11^C]AZT was converted to AZT-P after uptake in tumor cells but not in the blood.

Nucleoside transporters are involved in the cellular uptake of nucleoside analog antiviral drugs, as well as of nucleosides. In the present study, the addition of thymidine completely blocked the cellular uptake of [^11^C]AZT by C6 tumor cells, strongly indicating the specific involvement of nucleoside transporters (Fig. 7). In previous studies, several other transporters, including Na^+^-dependent concentrative nucleoside transporters (CNTs: human (h) /rat (r) CNT1 and hCNT3) [26, 27], Na^+^-independent equilibrative nucleoside transporters (ENTs), h/rENT2 [24], organic anion transporters (OATs) (h/rOAT1-3 and hOAT4) [28–32], and the organic cation transporter rOCT1 [33], were reported to be involved in AZT cellular uptake. Although the profile of nucleoside transporters in tumors has not yet been confirmed, these transporters may also contribute to the tumor-specific capacities of [^11^C]AZT uptake. Further studies are needed to identify the transporter(s) involved in [^11^C]AZT uptake.

Some studies have attempted to visualize the localization of viruses, such as HIV, within the cell using fluorescent proteins or fluorochromes in order to reveal the processes of cell invasion and virus particle maturation [34, 35]. It is well known that NRTIs such as AZT and d4T suppress the replication of HIV by inhibiting the activity of its reverse transcriptase [1, 2], indicating that [^11^C]AZT or [^11^C]d4T could have a role as a PET probe for imaging virus localization and virus dynamics in vivo. Further studies involving PET experiments and virus-infected animals are necessary to clarify virus localization and dynamics in vivo.

## Conclusions

We have assessed whether the proliferative activity of tumor cells can be visualized by PET analysis using [^11^C]AZT or [^11^C]d4T, and have demonstrated that [^11^C]AZT, but not [^11^C]d4T, is highly accumulated in proliferating tumor tissues compared to normal proliferating tissues. [^11^C]AZT might therefore be a useful PET probe for tumor imaging.

## Additional file

Additional file 1: Figure S1. PET images of C6 and HeLa tumor-bearing mice between 30 and 60 min after injection of 50 MBq of [^11^C]AZT (A) and [^11^C]d4T (B). Arrows and arrowheads indicate the C6 and HeLa tumors, respectively. **Figure S2** [^11^C]AZT and [^18^F]FDG uptake in aseptic inflammation model mice. Representative coronal PET images for [^11^C]AZT (A) and [^18^F]FDG (B) between 60–80 min after injection. Yellow dot circles indicated turpentine oil-induced inflammatory tissue. Biodistribution of [^11^C]AZT (C) and [^18^F]FDG (D) in turpentine oil-treated (black bar) and untreated (white bar) posterior thigh muscles. Data are means ± SD (*n* = 5, ****P* < 0.001). Histological analysis by hematoxylin and eosin staining of turpentine oil-untreated (E) and treated (F) muscles. Scale bar = 1 mm. **Figure S3** The accumulation levels in bone and tumor of [^11^C]d4T, [^11^C]AZT, and [^11^C]4DST. Thigh bones and tumors were collected after 80 min PET probe injection. Radioactivities in these tissues were measured by γ-well counter. The graph in (A) shows the %ID/g for tumor tissue and bone. Data are presented as means ± SD (*n* = 4 to 6). ***P* < 0.01 vs. [^11^C]AZT and [^11^C]d4T injected mouse. †*P* < 0.01 vs. [^11^C]d4T injected mouse. The graph in (C) shows the ratio of tumor-to-bone uptake of the labeled probes. **P* < 0.05 vs. [^11^C]4DST injected mice. ***P* < 0.01 vs. [^11^C]d4T injected mice.

## Abbreviations

AIDS: acquired immunodeficiency syndrome
AZT: Zidovudine
AZT-G: AZT-*β*-d-glucuronide
AZT-P: AZT-5′-monophosphate
d4T: Stavudine
HIV: human immunodeficiency virus
NRTI: nucleoside reverse transcriptase inhibitor
PET: positron emission tomography
TAC: time-activity curve
TLC: thin layer chromatography
