# Supplementary material for: A novel 11C-labeled thymidine analog, [11C]AZT, for tumor imaging by positron emission tomography
Source: EJNMMI Res. 2015 Sep 4;5:45. doi: 10.1186/s13550-015-0124-0 (PMC4597405; doi:10.1186/s13550-015-0124-0)
Supplement: Additional file 1: Figure S1. — PET images of C6 and HeLa tumor-bearing mice between 30 and 60 min after injection of 50 MBq of [11C]AZT (A) and [11C]d4T (B). Arrows and arrowheads indicate the C6 and HeLa tumors, respectively. Figure S2 [11C]AZT and [18F]FDG uptake in aseptic inflammation model mice. Representative coronal PET images for [11C]AZT (A) and [18F]FDG (B) between 60–80 min after injection. Yellow dot circles indicated turpentine oil-induced inflammatory tissue. Biodistribution of [11C]AZT (C) and [18F]FDG (D) in turpentine oil-treated (black bar) and untreated (white bar) posterior thigh muscles. Data are means ± SD (n = 5, ***P < 0.001). Histological analysis by hematoxylin and eosin staining of turpentine oil-untreated (E) and treated (F) muscles. Scale bar = 1 mm. Figure S3 The accumulation levels in bone and tumor of [11C]d4T, [11C]AZT, and [11C]4DST. Thigh bones and tumors were collected after 80 min PET probe injection. Radioactivities in these tissues were measured by γ-well counter. The graph in (A) shows the %ID/g for tumor tissue and bone. Data are presented as means ± SD (n = 4 to 6). **P < 0.01 vs. [11C]AZT and [11C]d4T injected mouse. †P < 0.01 vs. [11C]d4T injected mouse. The graph in (C) shows the ratio of tumor-to-bone uptake of the labeled probes. *P < 0.05 vs. [11C]4DST injected mice. **P < 0.01 vs. [11C]d4T injected mice. [file 13550_2015_124_MOESM1_ESM.doc]

**Supplemental FIGURE**


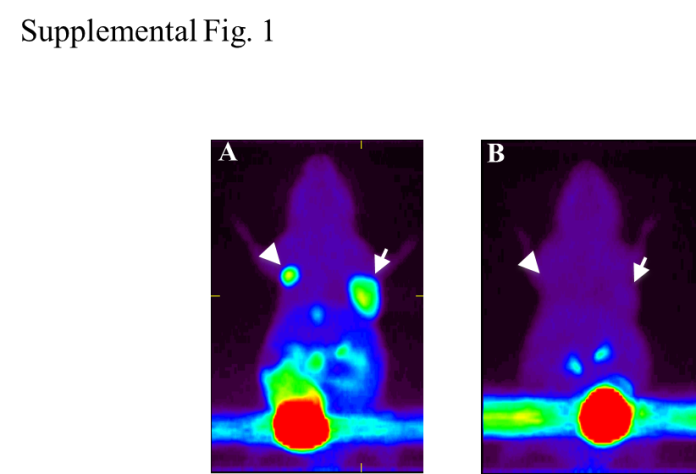


Supplemental FIGURE 1

PET images of C6 and HeLa tumor-bearing mice between 30 and 60 min after injection of 50 MBq of [^11^C]AZT (A) and [^11^C]d4T (B). Arrows and arrowheads indicate the C6 and HeLa tumors, respectively.


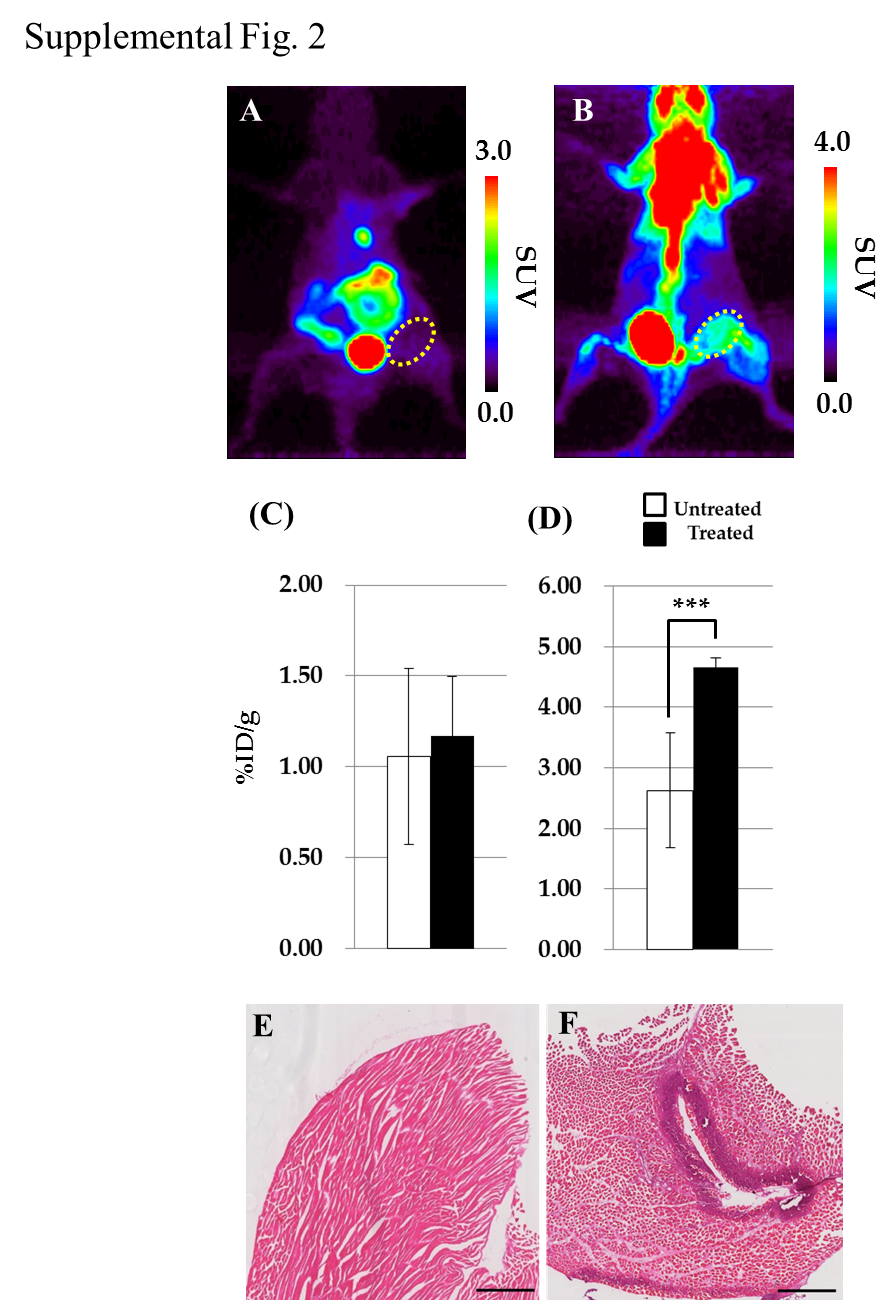


Spplemental FIGURE 2

[^11^C]AZT and [^18^F]FDG uptake in aseptic inflammation model mice. Representative coronal PET images for [^11^C]AZT (A) and [^18^F]FDG (B) between 60-80 min after injection. Yellow dot circles indicated turpentine oil-induced inflammatory tissue. Biodistribution of [^11^C]AZT (C) and [^18^F]FDG (D) in turpentine oil-treated (black bar) and untreated (white bar) posterior thigh muscles. Data are means ± S.D. (n=5, ****P* < 0.001). Histological analysis by hematoxylin and eosin staining of turpentine oil-untreated (E) and treated (F) muscles. Scale bar = 1 mm.


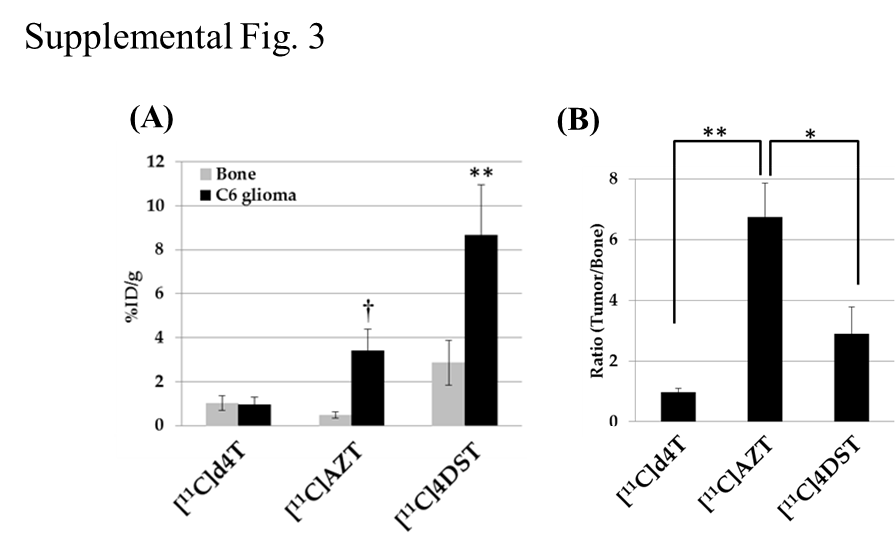


Supplemental FIGURE 3

The accumulation levels in bone and tumor of [^11^C]d4T, [^11^C]AZT and [^11^C]4DST. Thigh bones and tumors were collected after 80 min PET probe injection. Radioactivities in these tissues were measured by γ-well counter. The graph in (A) shows the %ID/g for tumor tissue and bone. Data are presented as means ± S.D. (n=4 to 6). **P < 0.01 vs. [^11^C]AZT and [^11^C]d4T injected mouse. †P < 0.01 vs. [^11^C]d4T injected mouse. The graph in (C) shows the ratio of tumor-to-bone uptake of the labeled probes. *P < 0.05 vs. [^11^C]4DST injected mice. **P < 0.01 vs. [^11^C]d4T injected mice.
